# Predictors of exercise-induced bronchoconstriction in subjects with mild asthma

**DOI:** 10.1186/s13223-021-00585-8

**Published:** 2021-08-14

**Authors:** Maroon Salameh, Laura Pini, Federico Quadri, Fabio Spreafico, Damiano Bottone, Claudio Tantucci

**Affiliations:** 1 Respiratory Medicine Unit, Spedali Civili, Brescia, Piazzale Spedali Civili 1 Italy; 2 Department of Clinical and Experimental Sciences, University of Brescia, Brescia, Italy

**Keywords:** Exercise-induced asthma, Dynamic hyperinflation, Airways hyperresponsiveness, Small airway disease

## Abstract

**Background:**

Physical effort is capable of triggering airway obstruction in asthmatics, the so-called exercise-induced bronchoconstriction in asthma (EIBa). This study was performed in subjects with mild persistent asthma, aiming to find predictors for developing EIBa.

**Methods:**

In 20 subjects with mild asthma, measurements of baseline functional respiratory parameters and airways responsiveness by a methacholine challenge were obtained on the first day. A maximal, symptom-limited incremental cardiopulmonary exercise test (CPExT) was performed the day after, with subsequent, repeated maneuvers of maximal full forced expiration to monitor the FEV_1_ change at 1,3,5,7,10 and 15 min after the end of the exercise.

**Results:**

19 subjects completed the two-days protocol. No functional parameters both at rest and during effort were useful to predict EIBa after stopping exercise. In asthmatics with EIBa, mean Inspiratory Capacity (IC) did not increase with increasing ventilatory requirements during CPExT because 6 of them (50%) displayed dynamic pulmonary hyperinflation (DH), as documented by their progressive increase of end-expiratory lung volume. This subgroup, showing earlier post-exercise FEV_1_ fall, had significantly lower forced mean expiratory flow between 25% and 75% of forced vital capacity (FEF_25-75%_) at rest (p < 0.05) and higher airways responsiveness, expressed as PD_20_FEV_1_ (p < 0.05) as compared with other asthmatics with EIBa.

**Conclusions:**

No functional respiratory parameters seem to predict EIBa in mild asthmatics. However, in those with EIBa, a subgroup developed DH during exercise, and this was associated with a baseline reduced forced expiratory flow rates at lower lung volumes and higher airway hyperresponsiveness, suggesting a prominent small airways impairment.

## Background

In patients suffering from bronchial asthma, physical exertion may acutely trigger airway obstruction leading to chest tightness, wheezing, dry cough, and dyspnea, that cease spontaneously with time or more quickly after treatment, namely bronchodilators with rapid onset of action [1].

This picture is now called exercise-induced bronchoconstriction in asthma (EIBa) [2], and although sometimes may be the prominent clinical manifestation of asthma, is enough to impair lifestyle and quality of life, especially among children, teens, young adults, and athletes [1, 3]. A high prevalence of asthmatics can be affected by EIBa [4–6].

Usually, airflow obstruction occurs soon after the end of exercise mainly due to both osmotic and thermal mechanisms [2, 7, 8], but sometimes may develop during exercise, limiting the subjects’ physical performance [9]. In this contest, an adequate treatment of the underlying airway inflammation and, if needed, a pre-treatment with fast short-acting (rarely fast long-acting) beta-2 selective agonists, or leukotriene-receptor antagonists is effective to control and prevent this event [10]. However, it would be useful to identify simple functional predictors of EIBa, mainly in asthmatic young people, in order to implement reliable strategies to avoid bronchoconstriction and related symptoms after or even during exercise, as much as possible. For this reason, the study aimed to identify functional parameters, either at rest or during exercise, predictors of effort-related airflow obstruction in subjects with persistent mild asthma suspected for EIBa.

## Methods

### Subjects

This prospective study was performed at the Respiratory Medicine Unit of Spedali Civili University Hospital of Brescia, Italy, from July 2018 to February 2019. To be included in the study, the subjects had to be aged 18–40 years and never smokers, have had a diagnosis of mild persistent asthma, according to GINA guidelines [11], supported by clinical judgment and objective measurements of lung function, including a positive methacholine challenge test for airway hyperresponsiveness and stable conditions for at least 8 weeks under usual treatment. Asthmatic subjects with other respiratory diseases and medical comorbidities were excluded from the study. The participants were recruited only if in the past they reported or were reported to have episodes suspected for effort-related acute respiratory symptoms.

### Study design

#### Functional respiratory tests

In the morning of the first day, after an 8-hour washout from short-acting bronchodilators, 48-hour washout from long-acting bronchodilators, and 4-week washout from any inhaled corticosteroids (ICS) or oral anti-cysteinyl leukotrienes, each subject performed in the first-day spirometry (BIOMEDIN Instruments, Padua, Italy) wearing a nose clip and breathing through a flanged mouthpiece. Slow vital capacity (VC) and inspiratory capacity (IC) were measured twice using a bell spirometer at rest in sitting position. At least three acceptable and reproducible maximal full expiratory maneuvers were performed to measure forced vital capacity (FVC), maximal expiratory volume in the first second (FEV_1_), and maximal forced expiratory flows ad different lung volumes.

Lung volumes were measured with a pressure-constant plethysmograph (BIOMEDIN Instruments, Padua, Italy). During the procedure, patients panted at a 0.7 Hertz frequency. Three acceptable tracings of mouth pressure versus box volume changes were averaged to achieve a final measurement of functional residual capacity (FRC). Total lung capacity (TLC) and residual volume (RV) were computed subsequently. In each circumstance, the best values were collected for analysis [12]. All tests were performed according to the ERS-ATS recommendations [13]. Predicted values of lung function parameters were those proposed by the European Community for Coal and Steel [14].

In the late morning, each subject performed a challenge test for measuring nonspecific, direct airway responsiveness using doubling doses of methacholine inhaled by dosimeter (MEFAR, Brescia, Italy). The tests were stopped just after more than 20% reduction of FEV_1_ from baseline (saline inhalation) to calculate airway sensitivity as PD_20_FEV_1_.

The second day, in the late morning, each subject underwent a symptom-limited, incremental cardiopulmonary exercise test by computer-driven cyclo-ergometer with the ramping protocol (15 watts increment per min) at a pedaling frequency of 50–60 per min (CPExT). This protocol always achieved 80–90 % of the predicted maximum heart rate towards the end of exercise and maintained it for few minutes. At rest and regular intervals of 2 min during exercise, the subjects were asked to inspire maximally toward total lung capacity (TLC) to obtain their inspiratory capacity (IC) that was measured off-line and expressed as % of TLC, at 33%, 66% and 99% of the peak workload.

At the end of the effort, repeated full forced expiratory maneuvers were requested to obtain maximal flow-volume curves to measure FEV_1_ at 1–3–5–7–10–15 min after stopping exercise. A FEV_1_ fall greater than 10% of FEV_1_ measured before starting exercise was considered as indicative of EIBa.

#### Statistics

Unless specified otherwise, data are expressed as the mean ± standard deviation. Comparing the functional variables between groups was performed according to the Student’s unpaired t-test or the Mann-Whitney U test if the normal distribution could not be assumed. Statistical significance was accepted if *p ≤ *0.05. Statistical analyses were performed using Graph Pad Prism 6.0 (Graph Pad Software, La Jolla, CA) and SPSS 23.00 (IBM, Armonk, NY).

## Results

Twenty asthmatic subjects were enrolled in the study. One subject refused CPExT and was excluded from the analysis. Among 19 subjects, 7 (37%) did not show EIBa with a ∆FEV_1_ max from baseline (made equal to 100) amounting to 97 ± 6% (absolute change − 2.6 ± 2.0%), while 12 (63%) had EIBa with a ∆FEV_1_ max from baseline (made equal to 100), amounting to 85±6% (absolute change − 16.2 ± 6.7%). Anthropometric and baseline functional data of the 19 patients of the study are shown in Table 1, all together and divided into 2 groups according to the presence or absence of EIBa. No significant differences emerged between the 2 groups. The mean duration of asthma was 11 ± 3 yr, with no significant difference between the two groups. All subjects were treated with inhaled corticosteroids that were withdrawn 4 weeks before the study and 7 (2 in the group without EIBa) with long-acting beta-2 agonists on top.

**Table 1:** Anthropometric and baseline functional respiratory parameters obtained for all asthmatic subjects and those divided according to the absence (non-EIBa) or presence (EIBa) of exercise-induced bronchoconstriction

	All	non-EIBa	EIBa	p
Subjects (n)	19	7	12	ns
Sex (m-f)	9-10	5-2	4-8	ns
Age (yrs)	27 ± 10	28 ± 8	27 ± 11	ns
Weight (kg)	68 ± 11	72 ± 13	66 ± 10	ns
Height (m)	1.71 ± 0.09	1.75 ± 0.09	1.69 ± 0.09	ns
BMI (kg/m^2^)	23 ± 4	23 ± 2	23 ± 5	ns
VC (% pred)	108 ± 8	107 ± 8	109 ± 9	ns
FVC (% pred)	109 ± 8	110 ± 8	108 ± 9	ns
IC (% pred)	100 ± 15	107 ± 11	96 ± 16	ns
FEV1 (% pred)	103 ± 8	105 ± 11	102 ± 7	ns
FEV1 /VC (% pred)	96 ± 7	97 ± 6	95 ± 8	ns
PEF (% pred)	96 ± 13	101 ± 13	94 ± 12	ns
FEF 25–75 % (% pred)	83 ± 20	88 ± 22	78 ± 19	ns
RV (% pred)	105 ± 32	100 ± 22	108 ± 36	ns
FRC (% pred)	108 ± 31	98 ± 25	113 ± 33	ns
TLC (% pred)	107 ± 11	104 ± 4	109 ± 13	ns
RV/TLC (% pred)	96 ± 21	94 ± 21	97 ± 22	ns
FRC/TLC (% pred)	100 ± 21	91 ± 19	105 ± 21	ns
PD_20%_FEV_1_ (mcg)	123 (33–683)	57 (40–273)	170 (33–683)	ns

*BMI* Body Mass Index, *VC* vital capacity, *FVC* forced vital capacity, *IC* inspiratory capacity, *FEV1* forced expiratory volume in first second, *FEV1/VC* tiffeneau index, *PEF* peak expiratory flow, *FEF 25–75%* forced mean expiratory flow-rates between 25% and 75% of FVC, *RV* residual volume, *FRC* functional respiratory capacity, *TLC* total lung capacity, *PD20%FEV1* cumulative dose at 20% fall of FEV1 from saline, geometric mean (range)

Mean data obtained at the peak of CPExT are presented in Table 2 with the IC values at different prefixed percent levels of the maximal effort, again in all subjects, and divided into 2 groups: those without EIBa, and those with EIBa. Not significantly different values of main variables of interest at peak exercise were found between the 2 groups. However, while mean IC progressively increased throughout exercise in asthmatics without EIBa, mean IC slightly decreased during exercise in asthmatics with EIBa.

**Table 2:** Parameters obtained during CPExT at the peak of exercise and values of IC at rest (bas.) and prefixed percentages of workload during exercise for all asthmatic subjects and those divided according to the absence (non-EIBa) or presence (EIBa) of exercise-induced bronchoconstriction

	All	non-EIBa	EIBa	p
Work peak (% pred)	104 ± 20	110 ± 15	100 ± 22	ns
VO2 peak (% pred)	99 ± 13	104 ± 15	96 ± 11	ns
HR peak (% pred)	91 ± 9	92 ± 11	90 ± 8	ns
VE peak (% pred)	53 ± 10	53 ± 12	52 ± 9	ns
RR peak (br/min)	35 ± 7	33 ± 6	37 ± 7	ns
Vt peak (L)	1.6 ± 0.4	1.6 ± 0.3	1.6 ± 0.5	ns
IC baseline (L)	3.04 ± 0.54	3.19 ± 0.66	2.95 ± 0.45	ns
IC 33% Work peak (L)	3.03 ± 0.56	3.28 ± 0.71	2.88 ± 0.40	ns
IC 66% Work peak (L)	3.02 ± 0.61	3.34 ± 0.81	2.82 ± 0.35	ns
IC 99% Work peak (L)	3.06 ± 0.72	3.46 ± 0.94	2.81 ± 0.44	ns

*VO2* oxygen consumption, *HR* heart rate, *VE* minute ventilation, *RR* respiratory rate, *Vt* tidal volume, *IC* inspiratory capacity

Data are mean ± SD

**Fig. 1:**
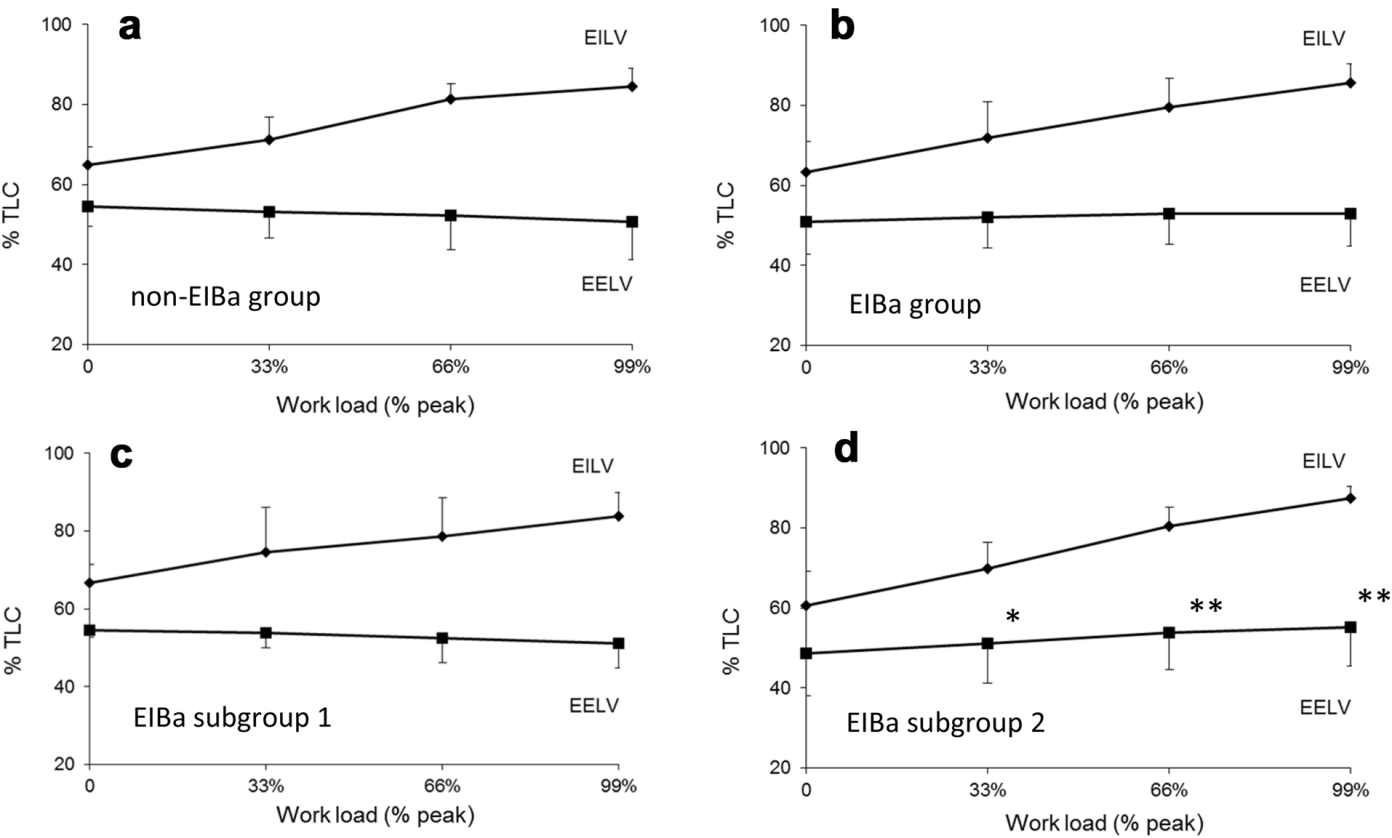
End-expiratory (EILV as % TLC; squares) and end-inspiratory (EELV as % TLC; diamonds) lung volume changes during exercise in asthmatics without EIBa (n = 7; **a**) and in asthmatics with EIBa (n = 12; **b**). EILV (squares) and EELV (diamonds) changes during exercise in asthmatics with EIBa without (n = 6; **c**) and with (n = 6; **d**) dynamic pulmonary hyperinflation. *p < 0.05, **p < 0.01 vs rest

The end-inspiratory (EILV as %TLC) and end-expiratory (EELV as % TLC) lung volume changes during exercise are illustrated in Fig. 1, panel a and b for the non-EIBa and EIBa groups, respectively, indicating a possible exertional dynamic pulmonary hyperinflation (DH) in asthmatics with EIBa. However, looking at the individual measurements of IC during exercise in asthmatics with EIBa, 6 out of 12 (50%) displayed a progressive, significant IC decrease and EELV (as %TLC) increase (EIBa subgroup 2), reflecting a noticeable DH. In contrast, the other 6 (EIBa subgroup 1) had normal lung mechanics behaviour during the effort, similar to that shown by the asthmatics without EIBa (see Table 3 and Fig. 1, panel d and c). The other different variable at the peak of exercise between the two subgroups with EIBa was the respiratory rate, much higher in the EIBa subgroup 2 (41.3 ± 6.5 vs 33.3 ± 6.4, p < 0.05).

**Table 3:** Values of IC at rest (baseline) and prefixed percentages of workload during CPExT in asthmatic subjects with EIBa, divided according to the absence (subgroup 1) or presence (subgroup 2) of exercise-induced pulmonary dynamic hyperinflation

	EIBa (subgroup 1)	EIBa (subgroup 2)
IC baseline. (L)	2.78 ± 0.44	3.09 ± 0.45
IC 33% Work peak (L)	2.81 ± 0.42	2.93 ± 0.41*
IC 66% Work peak (L)	2.87 ± 0.27	2.78 ± 0.43**
IC 99% Work peak (L)	2.95 ± 0.29	2.70 ± 0.53**

*IC* inspiratory capacity

Data are mean ± SD

*p < 0.05 and **p < 0.01 vs baseline

**Fig. 2:**
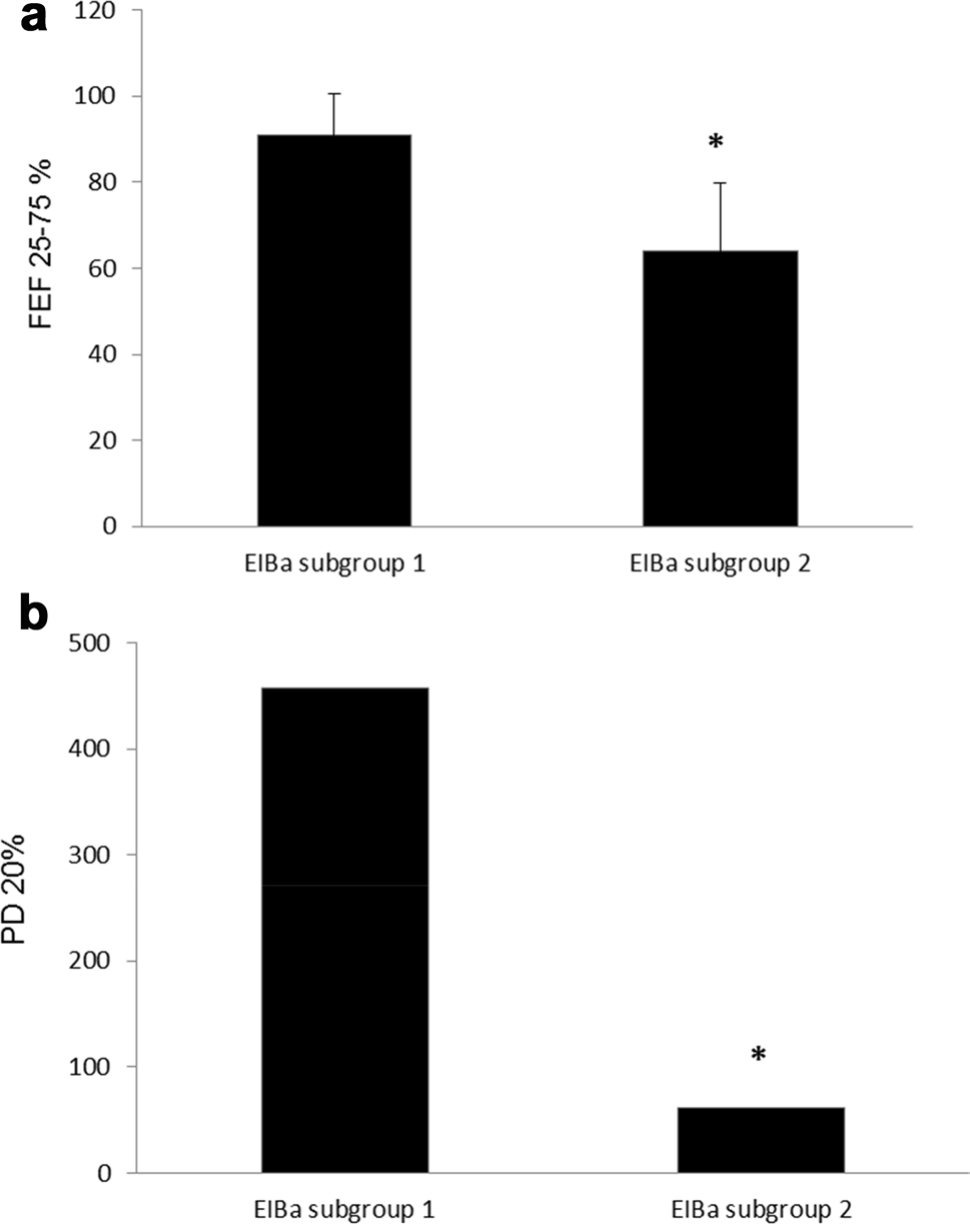
Mean Forced expiratory flow rates between 25 and 75% of FVC (FEF_25-75%_) at rest in asthmatics with EIBa, without (subgroup 1), and with (subgroup 2) dynamic pulmonary hyperinflation during exertion (panel a) * p < 0.05. The provocative dose of methacholine causing a 20% FEV_1_ fall from baseline (saline) FEV_1_ (PD_20_FEV_1_) in asthmatics with EIBa, without (subgroup 1), and with (subgroup 2) dynamic pulmonary hyperinflation during exertion (panel b) * p < 0.05. Geometric mean

By comparing the lung function measurements at rest between these two subgroups of asthmatics with EIBa, the only different parameter resulted in the maximal mean expiratory flow rate between 25% and 75% of forced vital capacity (FEF_25-75%_), which was significantly reduced in the subgroup with DH (EIA subgroup 2) (64 ± 16% pred. vs 91 ± 10% pred., respectively; p < 0.05), suggesting a greater structural involvement (inflammation/remodelling) of the small airways in these subjects (Fig. 2a). Patients with EIBa subgroup 2 showed significantly lower FEF25–75% values than patients without EIBa (64 ± 16% pred. vs 88 ± 22% pred., respectively; p < 0.05).

Interestingly, PD_20_FEV_1_ was markedly lower in the subgroup with EIBa and with DH (geometric mean 55 (33–261) mcg vs 389 (101–683) mcg; p < 0.05), partly explained by larger ventilation heterogeneity that is a marker of peripheral airway impairment (Fig. 2b). The PD_20_FEV_1_ value of the group without EIA was not different from the PD_20_FEV_1_ value observed in EIBa subgroup 2, amounting to 57 (40–273) mcg vs 55 (33–261) mcg, respectively. No differences were found between the non-EIBa group and EIBa subgroup 1 concerning baseline pulmonary function tests (including FEF_25-75%_) and parameters obtained during CPExT.

**Fig. 3:**
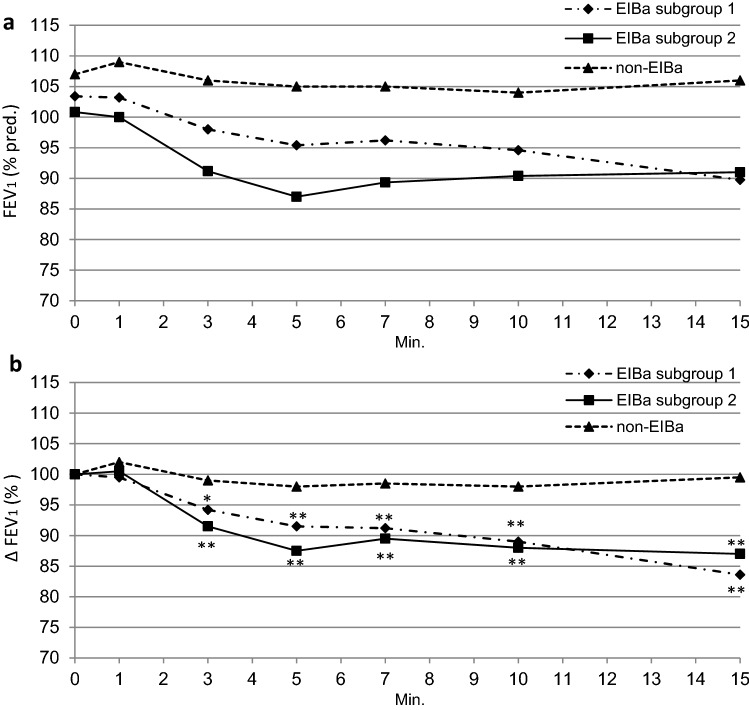
Changes of FEV_1_ both as absolute values (% pred.) (**a**) and as % change from baseline (∆FEV_1_ %) (**b**) after stopping exercise in asthmatics without EIA (n = 7) and with EIBa, without (n = 6; subgroup 1) and with (n = 6; subgroup 2) dynamic pulmonary hyperinflation during exertion (DH)

Finally, the post-exercise fall in FEV_1_, albeit more rapid and pronounced in the EIBa patients with DH during exercise (EIBa subgroup 2) as compared with those with EIBa without DH during exercise (EIBa subgroup 1), was not significantly different between them (Fig. 3).

## Discussion

The main findings of the study are the following. No functional parameter either at rest or during exercise was significantly different and able to distinguish in a population of subjects with mild persistent asthma who would have had EIBa and who not. In contrast with asthmatics without EIBa, IC did not increase during exercise in those with EIBa, and some of them (in our series 50%) developed DH during exercise with earlier FEV_1_ fall after stopping it. This subgroup of asthmatics with EIBa with DH during exercise (subgroup 2) showed a significant reduction of FEF_25-75%_ at rest and higher airway responsiveness to methacholine, suggesting a more extensive structural and functional involvement of small airways.

We did not find any respiratory functional parameters at rest, useful to predict the occurrence of exercise-induced bronchoconstriction in relatively young asthmatics with similar history and severity of disease (mild persistent asthma). Also, although patients developing EIBa had slightly lower mean peak values of workload and oxygen uptake during exercise, there were no significant differences in average between the two groups.

However, asthmatics with EIBa did not exhibit the expected EELV reduction during exercise (Fig. 1) with a number of them who developed DH, as witnessed by the progressive IC decrease and excessive RR increase at peak exercise (EIBa subgroup 2). These exertional mechanical constraints were associated with significantly reduced forced expiratory flows at lower lung volumes at baseline and higher airway responsiveness. In the presence of normal FEV_1_ and FVC, reduced FEF_25-75%_ strongly suggest an increased upstream resistance in these asthmatics’ small airways that, favouring a larger ventilation heterogeneity, may in part explain the higher airway hyperresponsiveness we observed concurrently [15].

The baseline reduced expiratory flow reserve at lower lung volumes, corresponding to the tidal volume range, may promote tidal expiratory flow limitation (EFL) when the ventilatory requirements increase during exertion. This possibility that is the occurrence of EFL was elegantly demonstrated by Kosmas and coworkers some years ago, using the negative expiratory pressure technique, in a large percentage of subjects suffering from asthma of different severity, from intermittent or mild to severe, but without EIBa, during symptom-limited incremental bicycle exercise test [16]. Interestingly, asthmatics who developed EFL during exercise had a lower baseline FEF_25-75%_ and FEF_75%_ than those without EFL. An increment of airway resistance throughout the exercise observed in some asthmatics [16, 17], especially with EIBa [9, 18, 19], can markedly increase this possibility. In these circumstances breathing at higher operative lung volumes allows to escape tidal EFL or have minimal EFL and continue exercising but at the expense of progressive DH [20].

Therefore, these indices such as FEF_25-75%_ and PD_20_FEV_1_, easily obtained at rest, could explain why some asthmatics with EIBa may have symptoms also during effort linked to the development of DH that might limit their exercise tolerance and maximal performance because of its associated mechanical constraints.

In the future would be interesting by using FOT to measure parameters of respiratory system resistance (Rrs5-Rrs20 difference) and reactance (Xrs5 or AX), exploring small and peripheral airway mechanical properties, to confirm this hypothesis in asthmatics suffering from EIBa who begin to have mechanical lung impairment and related symptoms also during effort [21]. Higher airway hyperresponsiveness (i.e., higher sensitivity as PD_20_FEV_1_) can ensue from deeper epithelial damage associated with either greater airway inflammation or abnormal neurogenic control or both, and this condition may certainly induce a worse functional impairment of small airways. Concurrently, larger ventilation heterogeneity sustained by a marked small airway involvement might also explain higher airway hyperresponsiveness [14]. We cannot discriminate between these two different mechanisms that, however, could coexist in this subgroup of asthmatics with EIBa.

This study is limited by the relatively small number of subjects and possibly by choice of the exercise challenge that is less than optimal for induction of EIBa, but needful to measure the operational lung volumes dynamic changes. However, we were able to elicit EIBa in more than 60% of the subjects recruited for suspicious exertional symptoms. Finally, we do not think that the absence of a matched control group could invalidate our findings’ meaning.

## Conclusions

None of the routine functional parameters measured at rest and during exercise can distinguish among mild asthmatics those who will develop EIBa or not. However, in some mild asthmatics with EIBa, dynamic hyperinflation can occur during exercise, possibly limiting their exercise tolerance and performance. Such as unfavourable mechanical constraint is associate with significantly decreased forced mean flow rates at lower lung volumes at rest and higher airway hyper-responsiveness, suggesting a more profound involvement of small airways in these subjects. That may explain why some asthmatics with EIBa become symptomatic during effort and not only after stopping it.

## Data Availability

All data generated or analyzed during this study are included in this published article.

## Abbreviations

EIBa: Exercise-induced bronchoconstriction in asthma
ICS: Inhaled corticosteroids
VC: Vital capacity
IC: Inspiratory capacity
FVC: Forced vital capacity
FEV1: Maximal expiratory volume in the first second
FRC: Functional residual capacity
TLC: Total lung capacity
RV: Residual volume
CPExT: Cardiopulmonary exercise test
EILV: End-inspiratory lung volume
EELV: End-expiratory lung volume
DH: Dynamic hyperinflation
FEF25-75%: Expiratory flow-rate between 25% and 75% of forced vital capacity
